# Supplementary material for: MetaRibo-Seq measures translation in microbiomes
Source: Nat Commun. 2020 Jun 29;11:3268. doi: 10.1038/s41467-020-17081-z (PMC7324362; doi:10.1038/s41467-020-17081-z)
Supplement: Supplementary file 10 — Supplementary Data 7 [file 41467_2020_17081_MOESM10_ESM.zip › File2/Confidence_High_Taxonomy/242715_out.krona.html]

Javascript must be enabled to view this page.

members
magnitude
magnitudeUnassigned
count
unassigned
taxon
rank

242715\_out

20

2
superkingdom
20

20
phylum
1239

20
class
186801

20
order
186802

family
20
186803

genus
20
841

2292066

SRS055017\_contig\_number\_2883
species
1

166486
18
species

SRS013940\_contig\_number\_3181SRS014412\_contig\_number\_contig-100\_6618.55739SRS014459\_contig\_number\_5664SRS018623\_contig\_number\_11145SRS043411\_contig\_number\_13613SRS044535\_contig\_number\_contig-100\_7081.7082SRS045739\_contig\_number\_8925SRS046068\_contig\_number\_contig-100\_4683.4684SRS056259\_contig\_number\_contig-100\_16227.16227SRS056519\_contig\_number\_18568SRS075341\_contig\_number\_23574SRS077641\_contig\_number\_21851SRS1055069\_contig\_number\_7400SRS143417\_contig\_number\_contig-100\_17748.104791SRS144714\_contig\_number\_25545SRS148721\_contig\_number\_12992SRS149075\_contig\_number\_contig-100\_4485.4486SRS893378\_contig\_number\_19179

2049040
1
species

SRS048164\_contig\_number\_contig-100\_16444.100682
